# Self-assembling ferritin nanoparticles coupled with linear sequences from canine distemper virus haemagglutinin protein elicit robust immune responses

**DOI:** 10.1186/s12951-021-01229-0

**Published:** 2022-01-10

**Authors:** Bo Wang, Shuang Li, Yongbo Qiao, Yu Fu, Jiaojiao Nie, Shun Jiang, Xin Yao, Yi Pan, Linye Zhao, Congmei Wu, Yuhua Shi, Yuhe Yin, Yaming Shan

**Affiliations:** 1 School of Chemistry and Life Science, Changchun University of Technology, Changchun, , 130012 Jilin China; 2 National Engineering Laboratory for AIDS Vaccine, School of Life Sciences, Jilin University, Changchun, 130012 Jilin China; 3 Changchun Xinuo BioTechnology Co., Ltd, Changchun, 130015 Jilin China; 4 Key Laboratory for Molecular Enzymology and Engineering, The Ministry of Education, School of Life Sciences, Jilin University, Changchun, 130012 Jilin China

**Keywords:** Canine distemper virus, Ferritin, Nanoparticles, Haemagglutinin, Linear sequence, Self-assembly, Epitope vaccine

## Abstract

**Background:**

Canine distemper virus (CDV), which is highly infectious, has caused outbreaks of varying scales in domestic and wild animals worldwide, so the development of a high-efficiency vaccine has broad application prospects. Currently, the commercial vaccine of CDV is an attenuated vaccine, which has the disadvantages of a complex preparation process, high cost and safety risk. It is necessary to develop a safe and effective CDV vaccine that is easy to produce on a large scale. In this study, sequences of CDV haemagglutinin (HA) from the Yanaka strain were aligned, and three potential linear sequences, termed YaH_3_, YaH_4_, and YaH_5_, were collected. To increase the immunogenicity of the epitopes, ferritin was employed as a self-assembling nanoparticle element. The ferritin-coupled forms were termed YaH_3_F, YaH_4_F, and YaH_5_F, respectively. A full-length HA sequence coupled with ferritin was also constructed as a DNA vaccine to compare the immunogenicity of nanoparticles in prokaryotic expression.

**Result:**

The self-assembly morphology of the proteins from prokaryotic expression was verified by transmission electron microscopy. All the proteins self-assembled into nanoparticles. The expression of the DNA vaccine YaHF in HEK-293T cells was also confirmed in vitro. After subcutaneous injection of epitope nanoparticles or intramuscular injection of DNA YaHF, all vaccines induced strong serum titres, and long-term potency of antibodies in serum could be detected after 84 days. Strong anti-CDV neutralizing activities were observed in both the YaH_4_F group and YaHF group. According to antibody typing and cytokine detection, YaH_4_F can induce both Th1 and Th2 immune responses. The results of flow cytometry detection indicated that compared with the control group, all the immunogens elicited an increase in CD3. Simultaneously, the serum antibodies induced by YaH_4_F and YaHF could significantly enhance the ADCC effect compared with the control group, indicating that the antibodies in the serum effectively recognized the antigens on the cell surface and induced NK cells to kill infected cells directly.

**Conclusions:**

YaH_4_F self-assembling nanoparticle obtained by prokaryotic expression has no less of an immune effect than YaHF, and H_4_ has great potential to become a key target for the easy and rapid preparation of epitope vaccines.

**Graphical Abstract:**

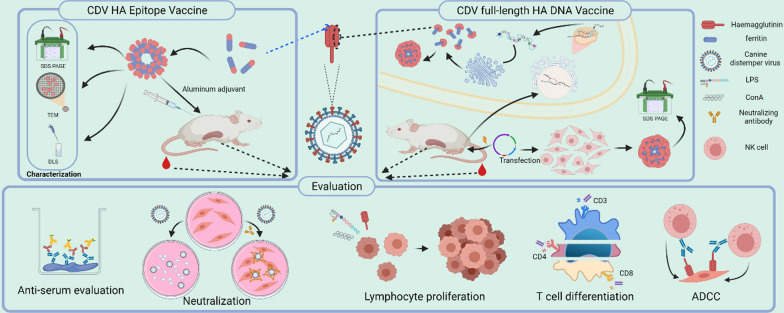

## Introduction

As a member of the genus Morbillivirus virus within the Paramyxoviridae family [1], canine distemper virus (CDV), which is transmitted through the respiratory tract, has expanded its hosts from dogs to a variety of terrestrial and aquatic predators [2–4]. The pandemic of canine distemper (CD) in wild animals and fur economic animals and the extremely high fatality rate of CD have caused great economic losses to related industries [5, 6].

Considering that the treatment of canine distemper may lead to immunosuppression and neurological sequelae [7], acquiring immunity to this pathogenic disease CDV is also necessary. Vaccination is still the best way to prevent infection and stop the spread of the virus. Live attenuated vaccines have replaced traditional inactivated vaccines, which have been abandoned because of poor protection. However, the impact of mass vaccination with live vaccines on wild species [8, 9] and the potential virulence of live vaccines for some animals are still concerns [10, 11]. These disadvantages increase the demand for CDV vaccines that ensure immune effects while taking into account the safety of vaccination. It has been confirmed that a strain isolated from Japan named Yanaka cannot cause infection in dogs but can still stimulate an effective immune response [12].

CDV consists of six structural proteins. Haemagglutinin protein (HA), as one of the surface glycoproteins of the virus, plays an important role in the process of infection [13–17]. It has been proven that the HA protein of CDV can effectively induce cellular immunity and humoral immunity against CDV [18–20]. The percentage of N-terminal glycosylation of the HA protein directly affects the intensity of CDV infection. In the process of infection, the HA protein first recognizes the receptor on the cell surface signalling lymphocyte activation molecule (SLAM) and then triggers a conformational change in the fusion protein (F). Both of them form oligomers to promote the virus genome to enter the cell to complete infection [21].

At present, to develop CDV vaccines with higher safety and better immune effects, studies are focused on multivalent viral vector vaccines. However, because this kind of vaccine involves the culture of eukaryotic cells and a vaccine with CDV as the virus vector cannot be secreted from the cell, the virus needs to be recovered [22, 23], which leads to a great increase in the production cycle and production cost. In comparison, subunit vaccines expressed in prokaryotic systems can greatly reduce the need for manpower and material resources. Epitope vaccines have become a new form of vaccine in recent years, especially in animal vaccines. However, considering that the target antigen selected by the epitope vaccine is short and the antigenicity is weak, an efficient delivery platform is needed. Self-assembled nanocarrier vaccines provide new ideas, such as ferritin, which is a cage protein of nonviral origin [24]. It can self-assemble into highly ordered 24 [25, 26] polymers and form multiple surfaces to display antigenic epitopes [27], which can greatly improve immunogenicity and enhance the efficacy of the vaccine while having low heterogeneity [28]. Some scholars have used ferritin in influenza vaccines [29], achieving good immune effects and universality. This provides the basis for ferritin as a nanovaccine carrier [30].

Therefore, in this study, using ferritin as the delivery vector, three prokaryotic systems expressing HA protein linear epitopes were constructed, and a eukaryotic expression vector expressing the full-length haemagglutinin protein was established to compare the immune effect of the epitope vaccine.

## Materials and methods

### Cells, virus and animals

Vero cells were obtained from BCHT Biotechnology Co. (China), and HEK-293T cells were obtained from the American Type Culture Collection (USA). Vero and HEK-293T cells were all cultured at 37 °C in an atmosphere of 5% CO_2_ in Dulbecco’s modified Eagle’s medium (Gibco, USA) containing 10% foetal bovine serum (Gibco) in a T-flask. The CDV strain CDV-11 used in this study was purchased from QILU ANIMAL HEALTH PRODUCTS Co., Ltd. (China). BALB/c mice (female, 6 weeks old, body weight: 18–20 g) were obtained from Liaoning Changsheng Biotechnology Co., Ltd. The animal trials in this study were carried out in accordance with the Regulations for the Administration of Affairs Concerning Experimental Animals approved by the State Council of the People's Republic of China (11–14-1988). All animal procedures were approved by the Institutional Animal Care and Use Committee of Jilin University (permit number: SCXK 2013-0001). The animal trials in this study were carried out in accordance with the Regulations for the Administration of Affairs Concerning Experimental Animals approved by the State Council of People's Republic of China (11–14-1988). All animal procedures were approved by the Institutional Animal Care and Use Committee (SYXK 2020-0018).

### Plasmid construction

The haemagglutinin amino acid sequence of the Yanaka strain (GenBank accession number BBB35231.1) was reverse-translated with eukaryotic codons and then synthesized into pet20b ( +) by GenScript (China). To successfully express the HA protein and increase its solubility, we deleted the transmembrane region of H (1–58). At the same time, by analysing the secondary structure, hydrophilicity, surface relative accessibility, polarity and fluidity of the protein sequence [31–35], we identified three potential immune cell linear epitopes with high scores (182–208, 373–420, 436–471) by screening. All the sequences were connected to ferritin (5–167) (NCBI accession no. WP_000949190) at the C-terminus and ended with a 6 × His tag. Then, the full-length HA carrying ferritin was cloned into the pSecTag2A vector by EcoR1 and Xho1 digestion sites, which also ended with 6 × His Tag. The ferritin-only sequence was obtained by PCR and cloned into pet20b ( +). All constructed plasmids were sequenced to express the desired protein sequence.

### Expression of target protein

The constructed prokaryotic expression plasmid was transformed into Transetta competent cells (TransGen Biotech, China). The bacteria were cultured to the logarithmic growth phase (OD value 0.6–0.8), and IPTG was added to a final concentration of 0.5 M for overnight expression at 16 °C.

Before transfection of the eukaryotic expression plasmid, HEK 393 T cells were inoculated in six-well plates at a density of 1 × 10^5^ per well and cultured overnight. The constructed plasmid was transfected into HEK-293T cells at a ratio of 1:4 (plasmid/PEI) with polyethyleneimine I (PEI). After 4 h of conversion, serum-free DMEM was used to replace the transfection mixture. Tissue culture supernatants containing unpurified YaHF protein were harvested three days after transfection.

### Purification and identification of protein

The sample solution containing the target protein was obtained by centrifugation, ultrasonic fragmentation and centrifugation. The target proteins were purified by affinity chromatography using Ni Focurose 6FF (IMAC) (HUIYAN Bio, China). After overnight dialysis, the purified protein was filtered and concentrated by 100 kDa Amicon Ultra-15 centrifugal filters (Merck Millipore Ltd). The final concentration of the protein was determined by a BCA protein assay kit (Beyotime, China). Finally, four target proteins denoted ferritin, YaH3F, YaH4F, and YaH5F were obtained and stored at − 80 °C.

The expression of all proteins was verified by using reducing sodium dodecyl sulfate–polyacrylamide gel electrophoresis (SDS–PAGE). The purified protein was used to verify the effect of ferritin self-assembly through 6% nonreducing SDS–PAGE, followed by blotting and then analysis by Western blotting using an anti-His tag monoclonal antibody (Invitrogen, Carlsbad, CA, USA). Western blotting was performed by using the ECL Plus substrate (Beyotime Biotech, Inc., China).

### Morphological detection of protein

To measure the particle size of the expressed protein, the sample was measured with a Zetasizer Nano ZS90 (Malvern Instruments, UK). Transmission electron microscopy (TEM) is one of the most powerful experimental means to study the expanded structure buried in the material [36]. To verify the assembly effect of ferritin, four kinds of proteins were characterized by TEM (Hitachi, Japan). The sample was dropped on a carbon-formvar copper grid, and negative staining was performed with phosphotungstic acid. Detection and observation were carried out under an accelerated voltage of 100 kV, and an image with a magnification of 40 K was obtained by using a charge-coupled device (CCD) camera system.

### Preparation of immunogen and immunity of mice

After a 7-day acclimatization period, 6-week-old female mice (*n* = 6 in each group) were immunized by intramuscular or subcutaneous injection 3 times on the 0th, 14th and 28th days. The protein was introduced by subcutaneous injection, and the nucleic acid was introduced by intramuscular injection. All the proteins were mixed with aluminium adjuvants at a 1:1 volume (15 μg, 200 μL/mouse) within two hours before immunization. The recombinant plasmid was diluted with phosphate-buffered saline (to 100 μg, (50 μL/mouse). To enhance the effectiveness of the nucleic acid immunization group, an electroporation device (Shanghai Teresa Biotech Co., Ltd., China) was used to assist the injection. Control mice were vaccinated with PBS. Serum samples were collected at 2-week intervals and heat-inactivated at 56 °C for 30 min prior to storage at − 80 °C until analysis.

### ELISA, antibody isotyping assay and cytokines assay

The serum titre was analysed by ELISA. Ninety-six-well plates were coated with synthetic polypeptide (GL Biochem Ltd, China) and haemagglutinin (YuduoBio Ltd, China) in PBS at 3 μg/mL and then incubated overnight at 4 °C. After three washes (0.05% Tween 20 in PBS), the wells were blocked with 3% bovine serum albumin (BSA) in PBS for 2 h, followed by another three washes. The treated serum was continuously diluted with PBS, added to the wells and incubated at 37 °C for 1.5 h. Then, the plates were washed 5 times, and an anti-mouse horseradish peroxidase (HRP)-conjugated antibody (Beijing Dingguo Changsheng Biotechnology Co., Ltd., China) was added and incubated at 37 °C for 45 min. The well was washed thoroughly with PBST, 3,3′,5,5′-tetramethylbenzidine solution was added to the wells, and colour development was stopped using 2 M H_2_SO_4_. The absorbance of plates was read at a wavelength of 450 nm using an iMarK™ Microplate Reader (Bio–Rad, USA). The reciprocal of the highest serum dilution resulting in absorbance 2.1 times the background value was considered to be the ELISA end point titre.

The antibody isotyping experiment was basically the same as the serum titre experiment. After coating ninety-six-well plates and blocking with 3% BSA/PBS, the serum was diluted and incubated at a fixed rate of 100 times. Anti-mouse IgG1, IgG2a, IgG2b, IgG3, IgA and IgM (Sigma–Aldrich, USA) were added to each well and incubated at room temperature (RT) for 2 h. Anti-sheep IgG alkaline phosphatase (Beijing Dingguo Changsheng Biotechnology Co., Ltd) was added to each well, and the secondary antibody was incubated at room temperature for 1 h. Then, p-nitrophenyl phosphate substrate diluted with AP buffer was added to each well at RT for 45 min. The reaction was terminated with 0.5 mM NaOH, and the absorbance was measured at a wavelength of 405 nm.

ELISA was also used to detect cytokines. Serum and splenocyte interleukin (IL)-2, IL-4 and interferon (IFN)-γ levels were detected by a mouse T-helper (Th) 1/Th2 ELISA Ready-SET-Go! Kit (eBioscience, USA). The single cell suspension was obtained by using a peripheral blood lymphocyte separation kit (Solarbio, China) and cultured with stimulants for 24 h. The concentration of cytokines was calculated by using the standard curve obtained from the measurement of the standard substance.

### Microneutralization assay

Vero cells were inoculated in 96-well plates and cultured overnight in DMEM containing 10% FBS. CDV-11 was serially diluted tenfold with virus medium (DMEM with 0.3% BSA). The treated diluent was added to the wells, and 8 compound wells were prepared. Cells added to the virus were incubated at 37 °C in 5% CO_2_ for 72 h. During this period, the visualization of the cytopathic effect on the cells was observed by optical microscopy and recorded, and then the Reed-Muench method was used to calculate the median tissue culture infective dose (TCID_50_) of the virus.

To determine serum neutralization antibody, Vero cells were inoculated into 96-well plates one night in advance. Twofold serial dilutions of the serum were prepared in virus medium and mixed with 100 TCID_50_ of CDV-11. First, the mixture was incubated at 37 °C in 5% CO_2_ for 1 h, and then the mixture was added to the cell plate under the same conditions. The neutralization titre of serum was calculated according to the cytopathic effect on the cells and expressed by the median inhibitory dose (ID_50_).

### Lymphocyte proliferation assay

After the mice were killed, the spleen was collected and ground, and lymphocytes were obtained. The cells were diluted to 1 × 10^6^ cells/mL with RPMI-1640 medium containing 10% FBS. The treated lymphocytes were inoculated into 96-well plates, and the corresponding stimulant (10 mg/mL) was added. Concanavalin A (ConA) (Sigma; 5 mg/mL) and lipopolysaccharide (LPS) were used as the positive controls, and RPMI-1640 medium was used as the negative control. After 48 h of stimulation, 3-[4,5-dimethylthiazol-2-y]-2,5-diphenyltetrasodium bromide tetrazolium (MTT; Sigma) was added and incubated for 4 h. Dimethyl sulfoxide was added to terminate the reaction. The average optical density was measured at a wavelength of 490 nm, and the stimulation index was calculated by the ratio of the growth of the experimental group to the growth of the negative group.

### Antibody-dependent cell-mediated cytotoxicity assay (ADCC) and detection of lymphocyte subsets

Haemagglutinin was coincubated with Vero cells (2 × 10^6^ cells/mL) for 2 h. The cells were stained with 5,6-carboxyfluorescein diacetate and succinimidyl ester (CFSE; Thermo Fisher) for 10 min at RT in the dark, labelling was stopped by adding cold complete RPMI-1640 medium, and the cells were incubated on ice for 5 min. Lymphocytes were obtained in the same way and diluted to 2 × 10^7^ cells/mL in RPMI-1640 medium. Treated Vero cells (100 μL) and serum diluted according to the titre (1 μL) were added to a 96-well plate and incubated at 37 °C for 15 min. Lymphocytes were added to each well and incubated at 37 °C for at least 2 h, and then 7-aminoactinomycin D (7-AAD; Thermo Fisher) was added to each well for staining. Data were collected by a Beckman Coulter Cytoflex S flow cytometer (United States) and analysed by CytExpert 2.0 software (Beckman Coulter).

Lymphocytes were obtained by the same treatment and diluted to 5 × 10^6^ cells/mL with cell staining buffer (Biolegend, USA). Trustain FcX™ PLUS (Biolegend, USA) was added at a proportion of 0.25 g to 100 μL of cells in the dark for 10 min and then stained with fluorescence-coupled antibodies FITC-CD3, PE-CD4, and APC-CD8 (Biolegend, USA) at 4 °C for 30 min in the dark. Similarly, data were collected by flow cytometry and analysed by CytExpert 2.0.

### Statistical analysis

**Scheme 1.**
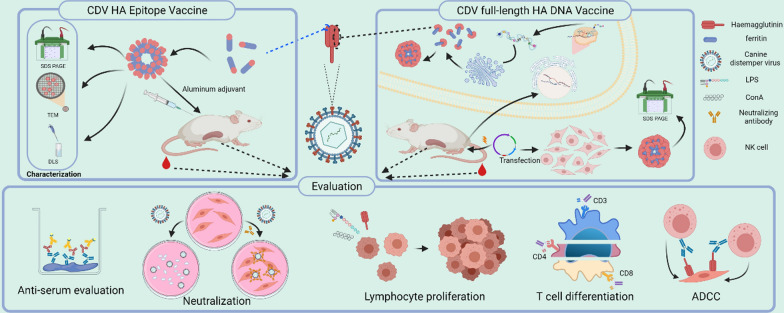
Schematic diagram of the immunogen designed by self-assembled nanoparticles ferritin was used to immunize in two ways and the subsequent immunoassay. Created with BioRender.com

Data are expressed as the mean ± standard deviation (SD). Statistical analyses were tested via Student’s t-test in Statistical Product and Service Solutions 25.0 software. Statistical significance was considered at *P < 0.05, **P < 0.01, and ***P < 0.001 (Scheme 1).

## Results

### Expression and characterization of target protein

**Fig. 1:**
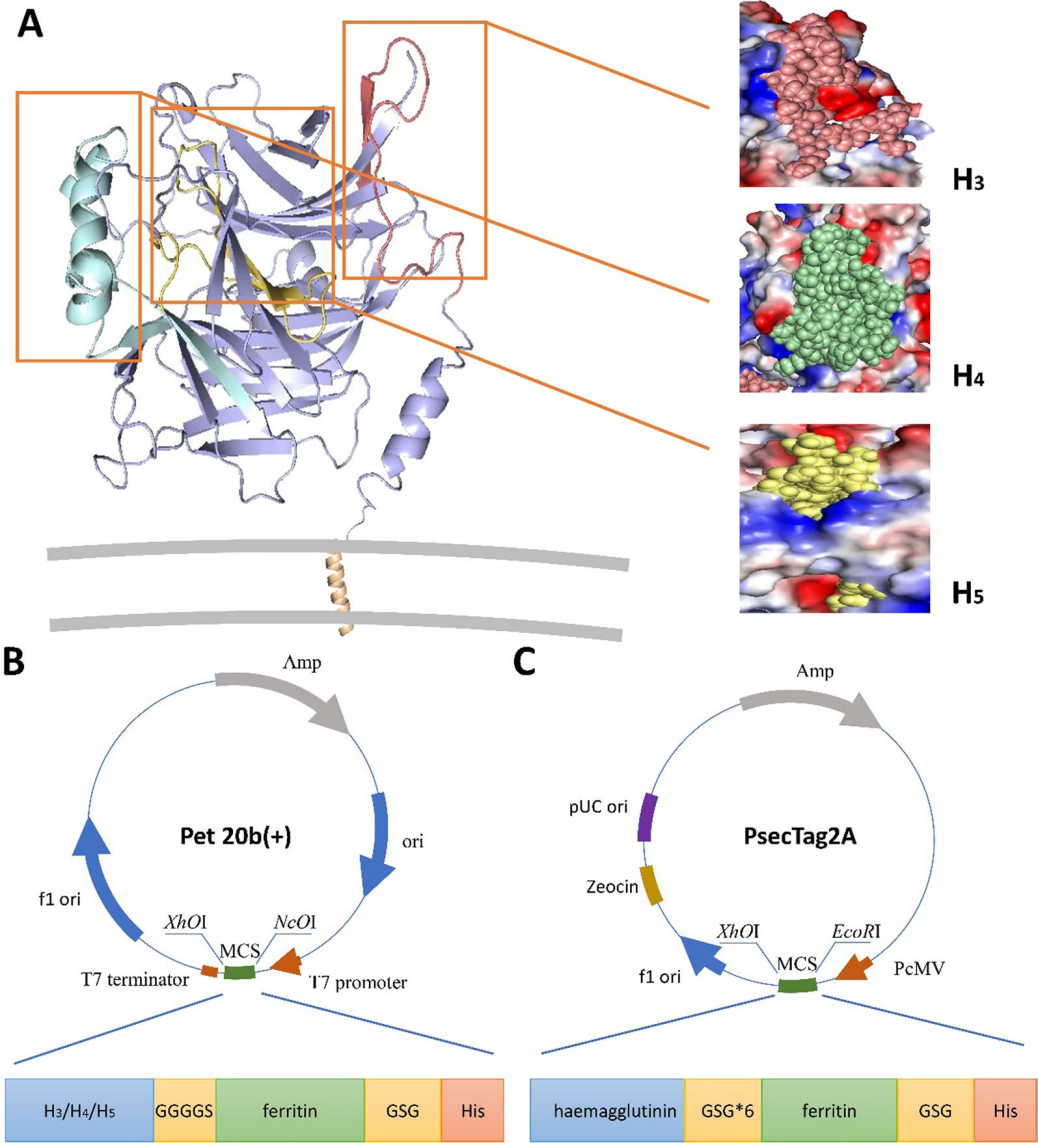
Immunogen constructs. **A** Mimic positions of the H_3_, H_4_ and H_5_ sequences on the haemagglutinin protein. **B** Construction of a prokaryotic expression system of antigenic epitopes. The ferritin sequence was ligated to the C-terminus of the epitope sequence by a linker. A His tag was added to the C-terminus of ferritin and inserted into pet20b ( +) between the *Nco*I and *Xho*I sites. **C** Schematic illustration of psecTag2A. Ferritin was connected to haemagglutinin in the same way, and a His tag was added to its C-terminus in psecTag2A between the *EcoR*I and *Xho*I sites

**Fig. 2:**
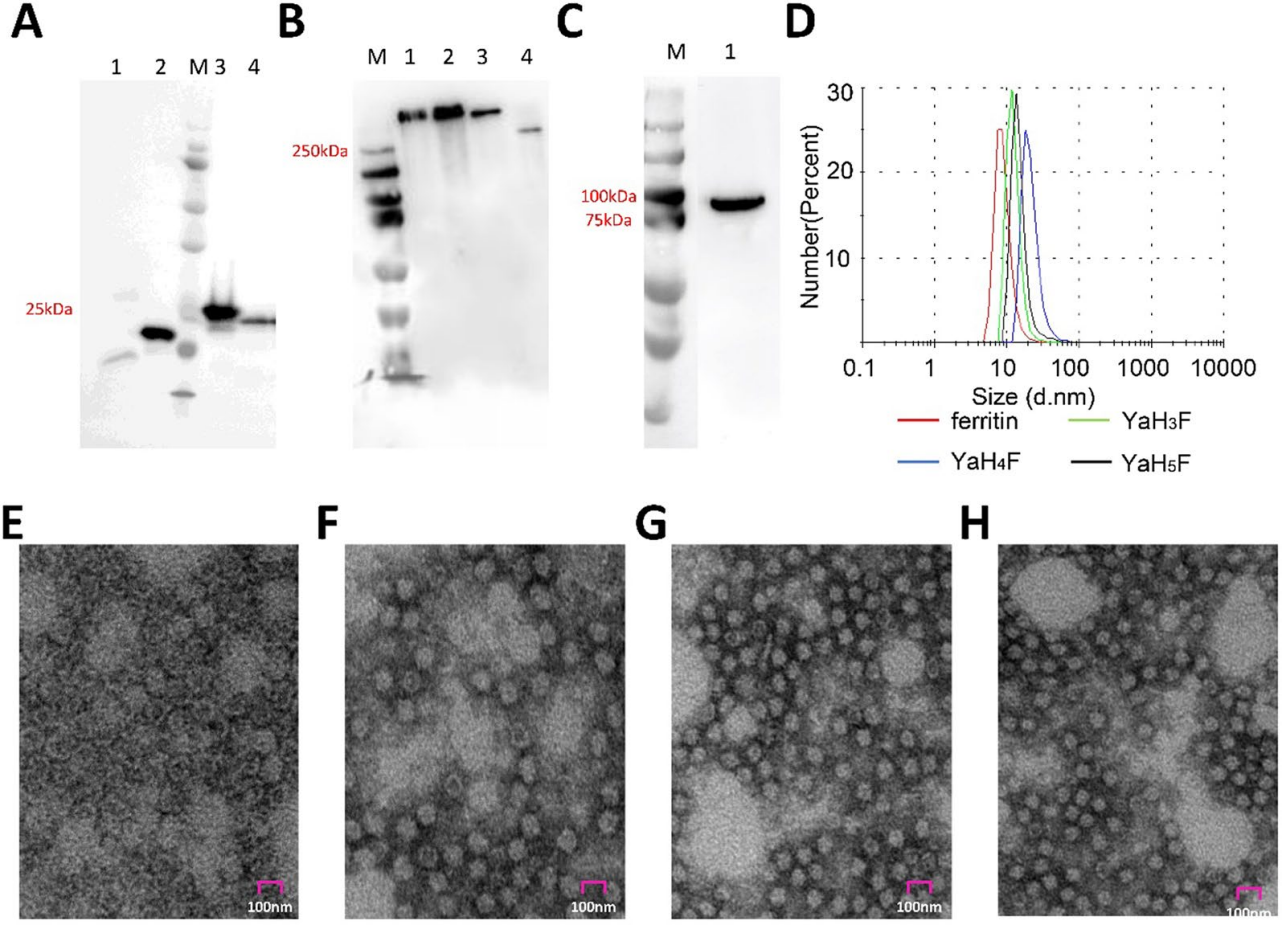
Characterization of target proteins. **A** Characterization of purified proteins and analysis by SDS–PAGE showing the molecular weights. Lane 1: ferritin; Lane 2: YaH_3_F; Lane 3: YaH_4_F; Lane 4: YaH_5_F. **B** Characterization of purified proteins and analysis by nonreducing SDS–PAGE showing the molecular weights. Lane 1: YaH_3_F; Lane 2: YaH_4_F; Lane 3: YaH_5_F; Lane 4: ferritin. **C** Western blot analysis of the plasmid transfection effect, Lane 1: YaHF. **D** Dynamic light scattering (DLS) of target proteins. Observations of ferritin nanoparticles by TEM (**E**), YaH_3_F (**F**), YaH_4_F (**G**), YaH_5_F (**H**)

The positions of three predicted potential immune determinants in haemagglutinin proteins were marked (Fig. 1A). Pet20b and pSecTag2A expression vectors were successfully constructed, as shown in Fig. 1B, C. To obtain the protein, the expression vectors were induced by IPTG and transfected with PEI, and then 6% nonreducing SDS–PAGE (Fig. 2B) and 10% reducing SDS–PAGE (Fig. 2A, C) were performed to verify the expression of the protein. The results showed that the plasmid transfected by PEI successfully expressed a single band, as expected (82 kDa). The purified and concentrated proteins produced a single band (> 250 kDa) higher than the molecular weight of the theoretical monomer (~ 25 kDa). This indicates that the expressed protein performs self-assembly.

The molecular weight of the assembled protein is too large (~ 600 kDa) for verification by nonreducing SDS–PAGE. Therefore, the effect of protein assembly was demonstrated and verified by particle size analysis (Fig. 2D) and TEM (Fig. 2E–H). The particle size of all ferritins carrying the target antigen is larger than that of unloaded ferritin, and the order of particle sizes formed by YaH_4_F, YaH_5_F and YaH_3_F is arranged according to the epitope size and consistent with the theoretical value. This suggests that the coupling of target epitopes does not affect the assembly of ferritin. All the samples had uniform morphology and formed hollow polygons, indicating that the expressed protein successfully completed its self-assembly.

### Specific antibody titre of immunized mice

**Fig. 3:**
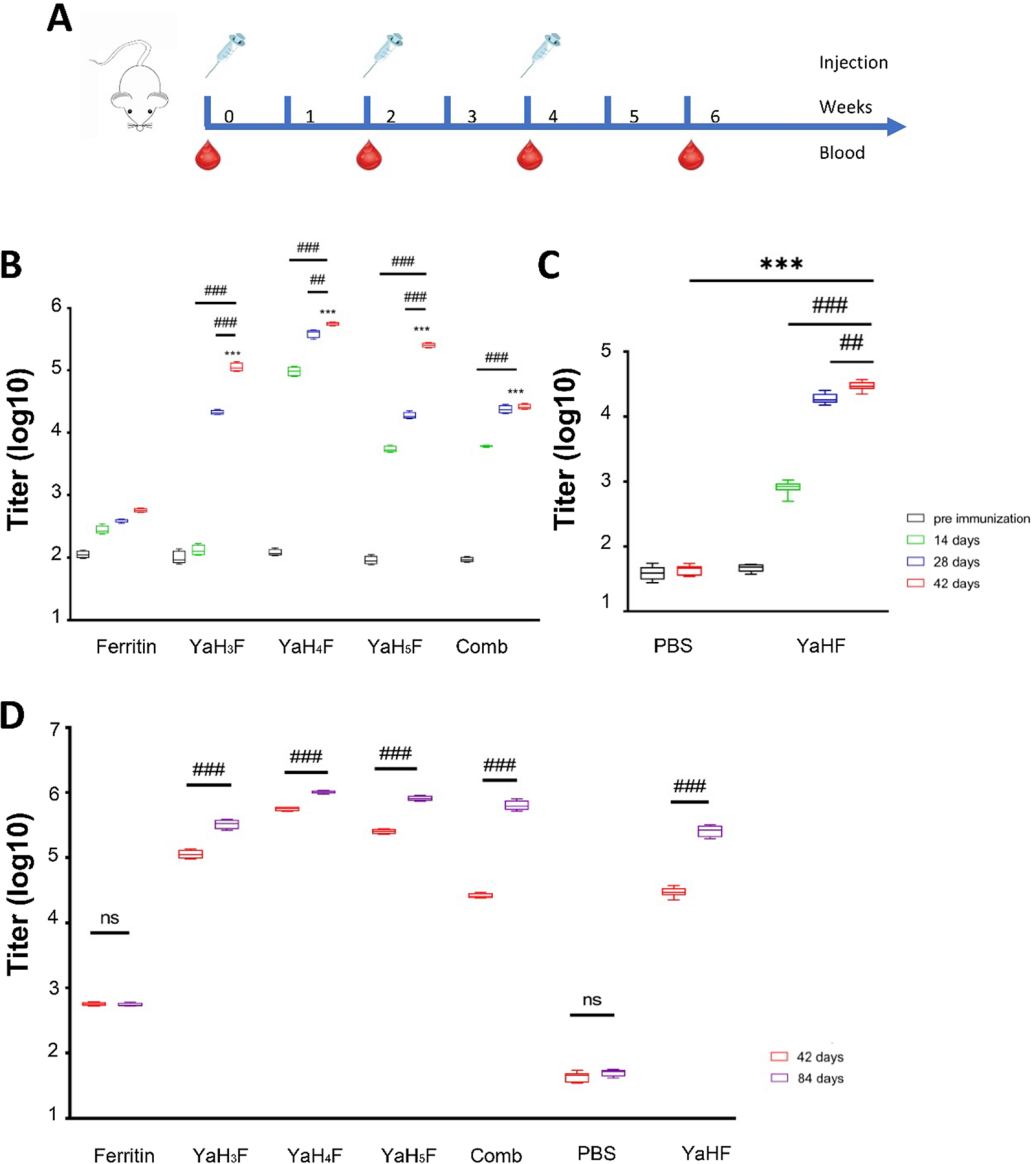
Evaluation of immunity and production of specific IgG in mice. **A** Mouse study design. The mice were immunized subcutaneously or muscularly at 0, 2 and 4 weeks, and blood was taken beforehand. Mice injected with ferritin and PBS were used as the negative control group. **B** Using commercially synthesized peptides as antigens, the titre of specific IgG in the serum was detected by ELISA. **C** The purchased haemagglutinin protein was used as the coating antigen, and the antibody titre in serum was detected by ELISA. **D** Synthetic peptides and commercial CDV haemagglutinin proteins were used as antigens to detect the serum titre by ELISA. Comb stands for combined immunization. ^##^*P* < 0.01, ^***^P < 0.001, ^###^*P* < 0.001

Five groups of BALB/c mice were immunized with ferritin, YaH_3_F, YaH_4_F, YaH_5_F and a mixture of the above three (combination) via hypodermic injection, and another two groups of BALB/c mice were immunized intramuscularly with PBS and YaHF. All mice were immunized three times on Days 0, 14, and 28; serum was obtained on Days 0, 14, 28 and 42 (Fig. 3A). The specific IgG titres of antisera were measured by ELISA. The results showed that all the protein immunogens produced high titre-specific immunoglobulins on the 28th day after immunization compared with the control group. It reached the highest level on the 42nd day and was significantly higher than that of the ferritin group (*p* < 0.001) (Fig. 3B). In terms of nucleic acid immunity (Fig. 3C), the titre of IgG in the YaHF group was significantly higher than before immunization within 0–14 days (*p* < 0.001) and increased to higher levels in 14–42 days. Furthermore, the titre of IgG in mice reached the peak at 28–42 days and was statistically significant compared with the value at 14–28 days (*p* < 0.001). Statistically, the H-specific IgG titre in the YaHF group was significantly higher than that in the PBS group at 28–42 days (*p* < 0.001). To detect the long-term effect of antibody in mouse serum, mouse serum was harvested on the 84th day after immunization, and the antibody titre was detected by the same method (Fig. 3D). The serum antibody titre of all experimental groups showed good long-term effects, and a significant improvement was still observed compared with 42 days without enhanced immunization (*p* < 0.001). These data showed that vaccination in all experimental groups effectively caused a high specific antibody response, and the antibody titre in the protein immunization group was higher than that in the nucleic acid immunization group. However, in view of the different coating antigens in the two tests, the immune effect needs to be verified by subsequent testing.

### Classified detection of antibody isotypes and detection of cytokines

**Fig. 4:**
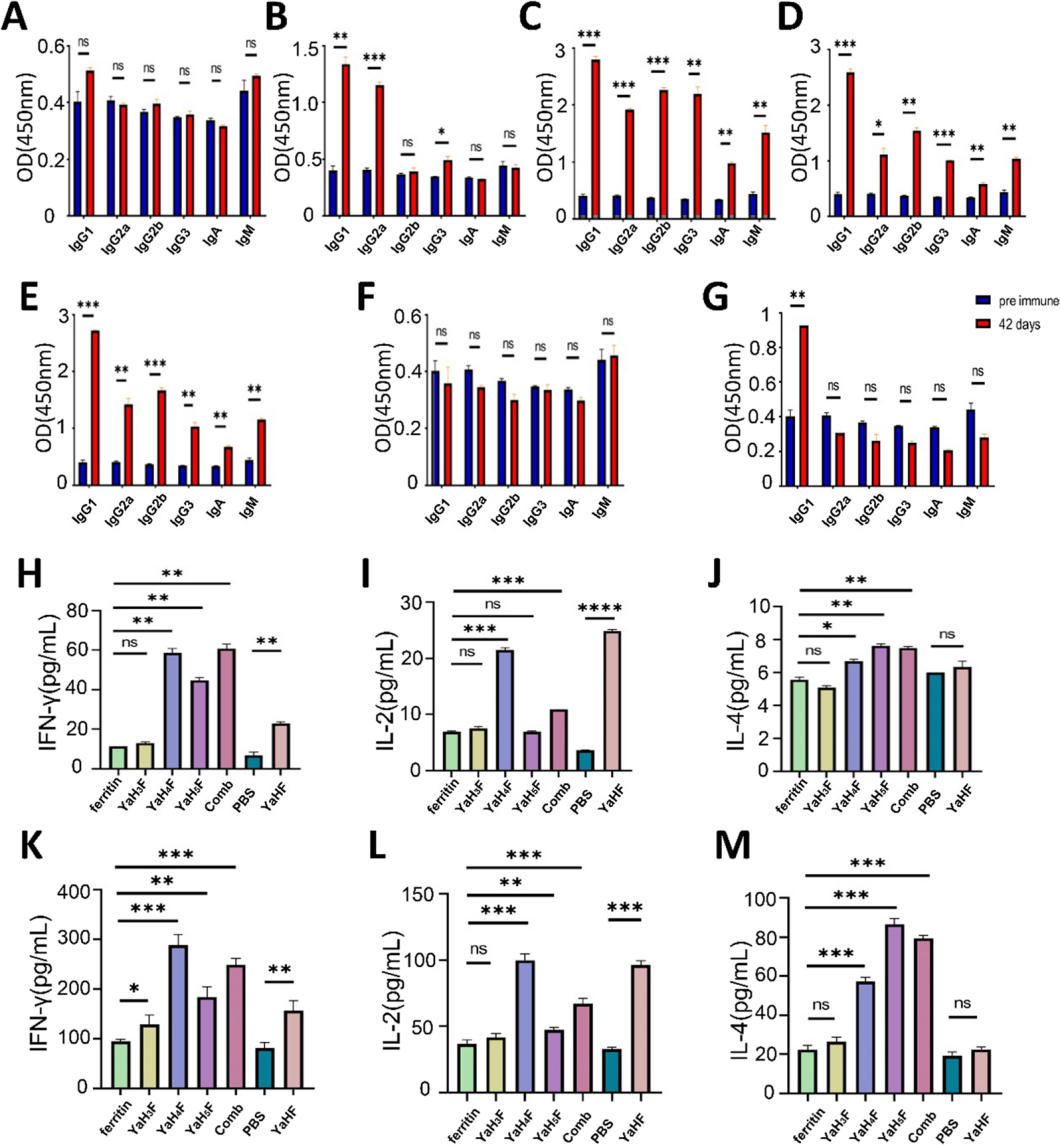
Antibody isotyping assay and cytokine detection. **A** Antibody isotyping assay of IgG1, IgG2a, IgG2b, IgG3, IgA and IgM isotypes elicited by ferritin; **B** YaH_3_F; **C** YaH_4_F; **D** YaH_5_F; **E** combination; **F** PBS; **G** YaHF. Detection of IFN-γ (**H**), IL-2 (**I**) and IL-4 (**J**) levels in antisera elicited by ferritin, YaH_3_F, YaH_4_F, YaH5F, combination immunity, PBS and YaHF. Detection of IFN-γ (**K**), IL-2 (**L**) and IL-4 (**M**) levels in cultured supernatant of spleen lymphocytes were cultured for 24 h with stimulator. Comb stands for combined immunization. ^*^P < 0.05, ^**^P < 0.01, ^***^P < 0.001, ^****^P < 0.0001

We used ELISA to detect different isotypes of immunoglobulin (IgG1, IgG2a, IgG2b, IgG3, IgA and IgM) to determine the main immune response and immune effect caused by immunogen (Fig. 4A–G). At 42 days after immunization, all the mice stimulated by YaH_4_F, YaH_5_F and the combination group produced significantly higher levels of all immunoglobulins tested than those before immunization (*p* < 0.05). Among them, the immune effect of YaH_4_F is particularly obvious. It is suggested that YaH_4_F may have a better immune effect in these three epitope vaccine groups. IgG1 and IgG2a are used as markers of the Th1 response and Th2 response, respectively, and their ratio reflects the state of the antibody response. Except for YaHF, all the other experimental groups induced an increase in IgG1 and IgG2a, and the level of IgG1 was significantly higher than that of IgG2a, indicating that the immunogen could induce both Th1 and Th2 type immune responses in mice with a higher likelihood of the Th2 type.

The IFN-γ, IL-2 and IL-4 assays via ELISA showed that the level of IFN-γ induced by YaH_4_F, YaH_5_F, the combined immunization group and YaHF was significantly higher than that in their respective control groups (*p* < 0.01) (Fig. 4H). In terms of the level of induced IL-2, only YaH_4_F, combined immunization and YaHF showed a significant increase (*p* < 0.001) (Fig. 4I). The level of IL-4 induced by YaH_4_F was higher than that of the control group (*p* < 0.05), while the level of IL-4 induced by YaH_5_F and combined immunization was significantly higher than that of the control group (*p* < 0.01) (Fig. 4J). Compared with the control group, YaH_3_F did not increase significantly in the detection of the three kinds of cytokines (*p* > 0.05). Compared with the cytokines in serum, the number of detectable cytokines in splenocyte increased significantly when stimulated. Similarly, in terms of splenocyte detection, there was no significant difference between YaH_3_F and the control group (*p* > 0.05) (Fig. 4L) (Fig. 4M), except for IFN-γ (*p* < 0.05) (Fig. 4K). The levels of three kinds of cytokines in YaH_4_F, YaH_5_F and combined immunization group were significantly higher than those in control group (*p* < 0.01).

### Neutralization effect of antibody on CDV-11 strain

**Fig. 5:**
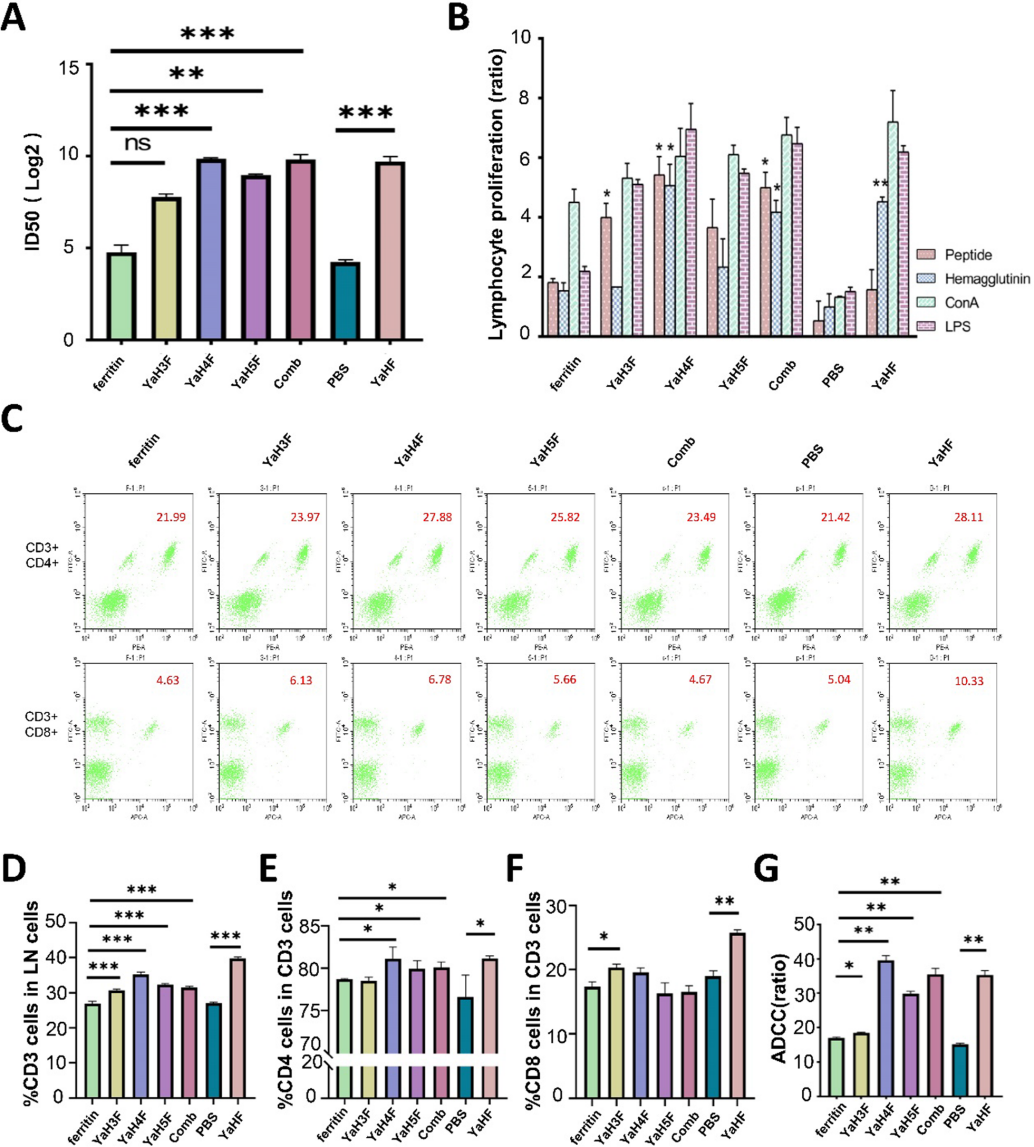
Detection of the titre of neutralizing antibody induced by self-assembled immunogen and the immune effect on lymphocytes. **A** The neutralization titre of the antibody was detected by the microneutralization method. Under the condition of the 100TCID_50_ CDV-11 strain, the half inhibitory dose of neutralizing antibody (ID_50_) was calculated. **B** Ferritin, YaH_3_F, YaH_4_F, YaH5F, combined immunization, PBS and YaHF induced lymphocyte proliferation stimulated by peptides, haemagglutinin, ConA and LPS. **C** Representative flow cytometric plots for measuring recruited and/or activated CD3/CD4 and CD3/CD8 in mice vaccinated with ferritin, YaH_3_F, YaH_4_F, YaH5F, combined immunization, PBS and YaHF. **D** Percentage of recruited and/or activated CD3 cells to lymphocytes. Differentiation degrees of CD4 from CD3 **E** and CD8 from CD3 **F** are shown. **G** The killing efficiency of antibody-dependent cell-mediated cytotoxicity (ADCC) compared with Triton X-100. Comb stands for combined immunization. ^*^P < 0.05, ^**^P < 0.01, ^***^P < 0.001

To verify the effectiveness of the antibodies produced by immunized mice, the serum of mice was detected by microneutralization. Immunization with YaH_4_F, combined immunization and YaHF produced the strongest neutralizing antibody, with a titre of more than 800 (*p* < 0.001) (Fig. 5A). Similarly, YaH_5_F also induced neutralizing antibodies with a titre of no less than 500 (*p* < 0.01). Although the antibody titre induced by YaH_3_F reached 220, it was not statistically significant (*p* = 0.2747).

### Spleen lymphocytes after immunization

Two stimuli, polypeptide and full-length haemagglutinin, were set up to evaluate the proliferative activity of mouse lymphocytes (Fig. 5B). When stimulated with ConA and LPS, all the experimental groups showed higher proliferation ability than the control group. The proliferation ability of lymphocytes stimulated by peptides in all epitope vaccine groups was stronger than that stimulated by haemagglutinin. In contrast, the proliferation of lymphocytes stimulated by haemagglutinin was stronger in the YaHF group. The proliferative response of spleen cells in mice immunized with YaH_4_F and in the coimmunization group was significantly higher than that in the ferritin group (*p* < 0.05). Simultaneously, lymphocyte proliferation in YaHF-immunized mice was significantly stronger than that in the PBS group (*p* < 0.01).

To investigate whether immunized mice recruited CD3 cells and to examine the differentiation of CD4 and CD8 in vivo, the spleen lymphocytes of mice 42 days after immunization were detected by flow cytometry. Representative flow cytometric plots for measuring recruited and/or activated CD3/4/8 are shown in Fig. 5C. Compared with the immune effect of ferritin and PBS, the number of CD3 cells activated by YaH_3_F, YaH_4_F, YaH_5_F, coimmunization and YaHF increased significantly (*p* < 0.001) (Fig. 5D). Among them, the level of CD4 subsets induced by YaH_4_F, YaH_5_F, combined immunization and YaHF was higher than that in the ferritin and PBS groups (*p* < 0.05) (Fig. 5E). In contrast, YaH_3_F induced higher CD8 subtypes (*p* < 0.05), and the growth effect of YaHF was significantly greater than that of PBS (*p* < 0.01) (Fig. 5F).

ADCC is an immune effect in which antibodies specifically recognize antigens and mediate the nonspecific killing of killer cells such as NK cells, and it is also one of the main immune methods. YaH_4_F, YaH_5_F, combined immunization and YaHF all mediated significant killing efficiency, ranging from 30 to 39% (*p* < 0.01) (Fig. 5G). The serum antibody of YaH_3_F-immunized mice also effectively enhanced the killing effect (*p* < 0.05).

## Discussion

CDV is an infectious pathogen with high morbidity and mortality, which has caused huge losses to the pet breeding and fur economy. Haemagglutinin protein is responsible for the binding of the virus to the receptor and for promoting the fusion of the virus, so it is used as the main target of CDV vaccine research. Commercial CDV vaccines have been weakened on the premise of safety. However, many studies have shown that there are still a large number of cases of canine distemper outbreaks in vaccinated animals [37]. Canine distemper is a disease for which acquired immunity can be lifelong [22], and it would be highly beneficial to develop a safer and more protective CDV vaccine.

In this study, ferritin was used as a nanocarrier to improve the efficacy of the vaccine, and three epitope proteins and a full-length eukaryotic expression plasmid were prepared. Through three immunizations in mice, the experimental data showed that full-length haemagglutinin carrying ferritin was successfully expressed in vivo. All experimental groups induced a significant increase in serum IgG antibody, which indicates a good humoral immune response, and the induced antibodies showed considerable long-term effects, which are very important for the prevention of CDV.

IFN-γ and IgG2a and the interleukins IL-4 and IgG1 reflect Th1 and Th2 cells, respectively. Generally, the activation of Th2 cells increases the production of IgG1 and inhibits the production of IgG2a activated by Th1 cells. All the experimental groups except YaHF increased the titres of IgG1 and IgG2a to varying degrees, and the ratio of IgG1 to gG2a was greater than 1, suggesting that all immunogens induce an immune response tending to Th2. In terms of antibody typing the content of IgA in serum is second only to that of IgG, and it exists widely in mucosal tissues such as the respiratory tract and digestive tract. High levels of IgA can effectively resist the invasion of viruses transmitted by the respiratory tract. Through the experiment, we found that both YaH_3_F, YaH_4_F and combined immunization can significantly increase the serum level of IgA, which will also provide an effective response in the initial stage of CDV immunization. Simultaneously, all three also induced an increase in IgM content. IgM can exert the function of cellular immunity by activating complements to dissolve bacteria and infected cells.

The production of the cytokine IFN-γ is mediated by Th1 cells involved in the antiviral action of cellular immune responses, while IL-4 is mediated by Th2 cells associated with humoral immune responses. YaH_4_F, YaH_5_F and combined immunization all increased the secretion of IFN-γ and IL-4 whether in serum or spleen cells, in contrast to YaHF, which induced only high levels of IFN-γ. IL-2 is synthesized mainly by CD4 + T cells stimulated by antigens, and IL-2 can also cause T cells to proliferate continuously, while IL-2 also acts on CD8 T cells to induce cytotoxicity. Both YaH_4_F and YaHF induced a strong increase in the level of IL-2, which can participate in both humoral and cellular immune responses. These results also suggest that several immunogens can induce humoral and cellular immune responses.

The titre of neutralizing antibody in serum is a more intuitive way to reflect the immune effect. The level of neutralizing antibody was detected by the CDV-11 strain at 100TCID_50_. The results showed that YaH_4_F, combined immunization and YaHF all resulted in high titres of neutralizing antibodies, which could exceed 800. This indicated that immunization with the three could effectively prevent infection by the CDV strain.

Lymphocytes are an important cellular component of the body's immune response and the main executor of almost all immune functions of the lymphatic system. The level of stimulated lymphocyte proliferation is an important index of cellular immunity. YaH_4_F and combined immunization induced a high level of proliferation under both stimuli, and when stimulated by ConA and LPS, the proliferation levels of both groups were significantly higher than those of the control group, suggesting that both the specific and nonspecific T lymphocyte proliferation responses of immunized mice had been enhanced. These results suggest that YaH_4_F and YaHF can improve the sustained cellular immune response in mice.

CD3 can transmit antigen information to the cell and initiate the intracellular activation process, which plays an important role in the early stage of immunity. All immunogens effectively caused an increase in CD3 in lymphocytes. CD3 differentiates into CD4 and CD8, which participate in immune regulation and direct killing in the immune process. The absolute count of CD4 and CD8 cells usually fluctuates greatly under different physiological conditions, while the ratio of CD4 to CD8 is relatively fixed, which can effectively reflect the immune response. When the ratio tends to increase, the body shows a state of sensitivity to antigens. After receiving antigen stimulation, T cells expressing CD4 can differentiate into Th1 or Th2 types due to the regulation of different immune factors and participate in different immune pathways. On the other hand, CD8 T cells usually differentiate into cytotoxic T lymphocytes after activation and specifically kill target cells. The results indicate that YaH_4_F, YaH_5_F and YaHF could increase the expression of CD4 on T cells and activate the corresponding immune pathway.

ADCC action is the nonspecific killing of infected cells combined with antibodies by killer cells. It is one of the mechanisms by which antibodies prevent or help to eliminate viral infection, and it can also reflect the ability of serum antibodies to specifically recognize antigens. The results showed that the ADCC ability of YaH_4_F, YaH_5_F, combined immunization and YaHF immunization was more than 1.5 times higher than that of the control group. It has been proven that the antibodies produced by the immunization of YaH_4_F, YaH_5_F and YaHF can effectively identify infected target cells and mediate the killing of lymphocytes to effectively avoid infection.

According to the above analysis, YaH_4_F protein for the formation of nanoparticles were successfully assembled and the plasmid YaHF that can be successfully expressed in vivo by immunization, the mice were induced to produce high titres of antibodies that had neutralizing activity and could play the role of ADCC. YaH_4_F and YaHF induced Th1 and Th2 immune responses and tended to induce Th1-type immune responses by synchronously increasing the production of IgG1 and IgG2a and high levels of IFN-γ. Simultaneously, immunization with YaH_4_F and YaHF significantly increased the proliferation of T lymphocytes and the level of immune stress in mice. However, the differentiation of T lymphocyte subsets indicated that their immune pathways tended to induce humoral immunity and cellular immunity. In conclusion, YaH_4_F and YaHF show a good immune response, and the H_4_ sequence can be used as a potential key antigen recognition site of CDV to prevent CDV more effectively.

However, considering that the immune effect of YaH_4_F is better than that of YaHF, a possible reason is that the glycosylation modification of proteins expressed in the eukaryotic system may not only trigger a specific immune response but also mask or overpackage some key antigenic determinants, thus affecting their immune effect. Similarly, the full-length haemagglutinin proteins also exhibit spatial folding, resulting in the entrapment of key epitopes. The epitope vaccine has higher safety than the traditional live attenuated vaccine. A large number of epitope vaccines have been tried on other pathogens and have good vaccine efficacy [38, 39]. In addition, the ability of epitope vaccines to address mutations in virus strains is incomparably better than that of traditional vaccines, especially for viruses whose wild strains have strong mutation abilities, such as CDV. The key factor in the immune effect of epitope vaccines is the selection of antigenic epitopes, which needs to be highly supported by bioinformatics data. To improve immune effectiveness and reduce the risk of immunopathological reactions, the ideal antigen target design should include as many neutralization sites as possible and reduce nonneutralization sites. In this experiment, three potential key epitopes of the CDV haemagglutinin protein were screened, and ferritin was used as a carrier to present the target antigen more efficiently. Aggregated nanoparticles can compensate for the lack of immunogenicity of epitope vaccines.

mRNA vaccines are a new type of vaccine that has undergone focused research and development in recent years and has been widely used to prevent COVID-19. The development of a canine distemper mRNA vaccine can not only produce the immune effect described above but also has a simple production process with no need for cell culture, low cost and no need for mRNA to enter the nucleus, and there is no risk of integration into the host genome. Similarly, as the main host of respiratory infectious diseases in dogs and some economic animals, it is very necessary to form immunity against virus attacks in their bodies, which will be testified in follow-up experiments.

## Conclusion

According to the experimental results, the H_4_ epitope had good antigenicity and immunogenicity, which would render YaH_4_F an attractive vaccine candidate for the prevention and control of CDV infection. In addition, the efficient self-assembly ability of ferritin enables it use as a potential presentation carrier for epitope targets.

## Data Availability

The datasets generated and/or analysed during the current study are available in the [GENE] repository, [https://www.ncbi.nlm.nih.gov/gene/].

## Abbreviations

CDV: Canine distemper virus
HA: Haemagglutinin
SLAM: Signalling lymphocyte activation molecule
SDS–PAGE: Sulfate–polyacrylamide gel electrophoresis
TEM: Transmission electron microscopy
CCD: Charge-coupled device
BSA: Bovine serum albumin
HRP: Horseradish peroxidase
RT: Room temperature
IFN: Interferon
TCID50: Tissue culture infective dose
ID50: Inhibitory dose
ConA: Concanavalin A
LPS: Lipopolysaccharide
MTT: 3-[4,5-Dimethylthiazol-2-y]-2,5-diphenyltetrasodium bromide tetrazolium
ADCC: Antibody-dependent cell-mediated cytotoxicity assay
7-AAD: 7-Aminoactinomycin D
